# Lymphomatosis Cerebri Presenting as Steroid-Refractory Demyelinating Disease: A Diagnostic Challenge

**DOI:** 10.7759/cureus.106611

**Published:** 2026-04-07

**Authors:** Genesis G Koodaly, Akshay Baheti, Suyash Kulkarni, Lingaraj Nayak, Vasundhara Patil

**Affiliations:** 1 Department of Radiology, Tata Memorial Hospital, Mumbai, IND; 2 Department of Medical Oncology, Tata Memorial Hospital, Mumbai, IND

**Keywords:** diffuse white matter disease, lymphomatosis cerebri, magnetic resonance imaging, primary central nervous system lymphoma, stereotactic brain biopsy, steroid-refractory demyelination, white matter infiltration

## Abstract

Lymphomatosis cerebri (LC) is a rare infiltrative variant of primary central nervous system lymphoma characterized by diffuse cerebral involvement without formation of a discrete mass lesion, which can mimic inflammatory demyelinating disease on imaging. We report a diagnostically challenging case of a 31-year-old man who presented with progressive bilateral visual impairment and diffuse white matter abnormalities initially managed as a demyelinating disease. Magnetic resonance imaging demonstrated widespread bilateral T2-weighted and fluid-attenuated inversion recovery hyperintensities involving the optic pathways, deep and periventricular white matter, basal ganglia, thalami, brainstem, and cerebellar peduncle, with minimal contrast enhancement and mild diffusion restriction. The patient failed to improve despite treatment with high-dose corticosteroids and other immunomodulatory therapies and subsequently developed seizures with progressive neurological deterioration. Extensive laboratory and cerebrospinal fluid evaluation did not support an inflammatory, infectious, or autoimmune etiology. Stereotactic brain biopsy performed due to clinical progression revealed diffuse angiocentric infiltration by malignant lymphoid cells, confirming the diagnosis of LC. This case highlights the potential for LC to masquerade as steroid-refractory demyelinating disease and underscores the importance of recognizing suggestive imaging patterns that should prompt early consideration of brain biopsy.

## Introduction

Primary central nervous system lymphoma (PCNSL) is an uncommon extranodal non-Hodgkin lymphoma confined to the brain, leptomeninges, spinal cord, or eyes [1]. It most often presents radiologically as a focal, avidly contrast-enhancing mass lesion with restricted diffusion and is typically of diffuse large B-cell lineage [1-4]. A rare, less recognized variant of PCNSL is lymphomatosis cerebri (LC), characterized by diffuse infiltration of cerebral white matter without formation of a discrete mass [3]. There are only a limited number of cases reported in the literature. It predominantly affects older adults; in an analysis of seven reported cases, the median age at presentation was 58 years [5]. The term lymphomatosis cerebri was first proposed by Bakshi et al. to describe a diffuse infiltrative pattern of PCNSL that closely mimics gliomatosis cerebri on imaging [6]. Gliomatosis cerebri, however, is now considered an outdated term and is currently classified under diffuse infiltrating gliomas in the World Health Organization classification.

Unlike classical PCNSL, LC demonstrates a distinct imaging phenotype, most commonly manifesting as widespread T2-weighted and fluid-attenuated inversion recovery (FLAIR) hyperintensities with minimal or absent contrast enhancement and limited diffusion restriction [7,8]. This pattern closely overlaps with inflammatory demyelinating disorders and infiltrative gliomas, frequently leading to diagnostic uncertainty and delayed recognition of the underlying malignancy [7-9]. Although relative diffusion restriction may occasionally be observed, and lesions can demonstrate homogeneous hypermetabolism on fluorodeoxyglucose-positron emission tomography (FDG-PET) compared to gliomatosis cerebri, definitive diagnosis frequently requires histopathological confirmation [10].

The diagnostic challenge of LC is further compounded by its frequent initial response to corticosteroid therapy, which may transiently reduce imaging abnormalities while obscuring histopathological features, thereby delaying tissue diagnosis [2,11]. Consequently, LC is often misdiagnosed as inflammatory demyelinating disease, resulting in prolonged empiric immunosuppression and deferred definitive treatment [9].

We report a case of LC in a young adult who presented with progressive visual impairment and diffuse white matter abnormalities initially interpreted as a demyelinating disease. This case emphasizes the importance of recognizing imaging features suggestive of LC and maintaining a high index of suspicion in steroid-refractory diffuse white matter disease.

## Case presentation

A 31-year-old man with no significant past medical history presented in April 2025 with acute-onset blurring of vision in the right eye, predominantly affecting the nasal field, which progressively worsened over several days and subsequently involved the left eye. He was initially evaluated at an outside facility and diagnosed with right optic neuritis. He also reported intermittent low-grade fever. There was no history of seizures, altered sensorium, focal neurological deficits, or known systemic malignancy at presentation.

Initial magnetic resonance imaging (MRI) demonstrated involvement of the bilateral optic nerves and optic chiasm, along with thickening of the left optic tract and multiple T2-weighted/FLAIR hyperintense lesions in the juxtacortical and periventricular regions of the bilateral frontoparietal lobes. Based on a presumed demyelinating etiology, the patient was treated with intravenous methylprednisolone (1 g/day for five days), followed by oral steroids (40 mg daily for 15 days, tapered to 20 mg daily for an additional 15 days). However, no clinical improvement was observed. He was subsequently readmitted and treated with intravenous immunoglobulin at a total dose of 2 g/kg over five days.

**Figure 1:**
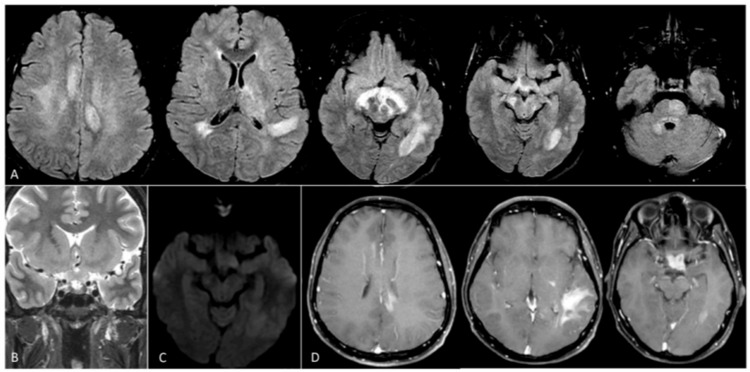
Magnetic resonance imaging of the brain. Fluid-attenuated inversion recovery images (A) show ill-defined heterogeneous hyperintense signals involving the bilateral periventricular white matter, centrum semiovale, posterior limb of the left internal capsule, left basal ganglia and thalamus, right middle cerebellar peduncle, pons, and left optic radiation, reflecting extensive multiregional involvement, with no associated mass effect. Thickening of the optic chiasma with abnormal T2 hyperintense signal (B) and mild restricted diffusion is noted (C). Patchy areas of abnormal enhancement are seen in these regions, including the optic chiasma and tracts (D).

Follow-up MRI performed on May 28, 2025, demonstrated diffuse thickening of the bilateral optic nerves with marked involvement of the optic chiasm, optic tracts, and optic radiations extending into the bilateral occipital lobes, more pronounced on the left (Figure 1). Additionally, multiple ill-defined T2-weighted and FLAIR hyperintense lesions were noted involving the bilateral corona radiata, centrum semiovale, posterior limbs of the internal capsules, periventricular and juxtacortical white matter, and bilateral parieto-occipital regions. Similar signal abnormalities were also seen in the deep gray nuclei, including the left basal ganglia and thalamus, as well as the brainstem (midbrain and pons) and right middle cerebellar peduncle. These lesions demonstrated minimal contrast enhancement and mild diffusion restriction. There was no mass effect seen.

Given the imaging appearance and clinical presentation, the patient was treated for inflammatory demyelinating disease with intravenous methylprednisolone followed by oral corticosteroids, without clinical improvement. Further investigations were performed to exclude infectious, autoimmune, and demyelinating etiologies.

Laboratory investigations revealed leukocytosis, low hemoglobin, and normal platelet counts. Cerebrospinal fluid (CSF) analysis showed elevated protein, near-normal glucose, lymphocytic predominance (5 cells/mm³), and absence of oligoclonal bands (Table 1). CSF cytology and flow cytometry were negative for malignant cells. Microbiological studies, including bacterial cultures, acid-fast bacilli, cryptococcal antigen, and Venereal Disease Research Laboratory, were negative. Autoimmune workup, including antinuclear antibody blot, aquaporin-4 (AQP4), and myelin oligodendrocyte glycoprotein (MOG) antibodies, was also negative. Visual evoked potentials were unremarkable.

**Table 1:** Laboratory results with normal ranges. WBC: white blood cell; RBC: red blood cell; CSF: cerebrospinal fluid

Investigation	Result	Normal range
Hemoglobin	11.3	13.0–17.0 g/dL
RBC count	3.46	4.5–5.5 × 10^12^/L
Platelets	294	150–400 × 10^9^/L
WBC count	16.3	4.0–10.0 × 10^9^/L
CSF protein	123.2	15–45 mg/dL
CSF glucose	71.0	40–70 mg/dL

Given persistent symptoms, the patient underwent five cycles of plasma exchange and received rituximab (1 g), while continuing oral steroids (30 mg daily). Despite aggressive immunomodulatory therapy, his condition worsened, and he developed right focal status epilepticus with altered sensorium and decreased responsiveness.

In view of progressive neurological deterioration and diffuse imaging abnormalities, a stereotactic brain biopsy was performed from a right parietal lesion. Histopathological examination revealed perivascular cuffing and diffuse parenchymal infiltration by large atypical lymphoid cells. Immunohistochemistry demonstrated strong CD20 positivity, confirming B-cell lineage, with a Ki-67 proliferation index of approximately 20-25%.

Whole-body FDG-PET/CT performed after histopathological diagnosis showed no evidence of systemic lymphoma, confirming primary central nervous system involvement. In the absence of a discrete mass lesion and with diffuse white matter infiltration, the findings were consistent with LC.

The patient was initiated on induction chemotherapy as per the CALGB MTR protocol (methotrexate, temozolomide, and rituximab). High-dose methotrexate was administered on July 13, 2025, followed by rituximab and temozolomide as per protocol. Despite treatment, his neurological status continued to decline, complicated by non-convulsive status epilepticus and recurrent microaspiration. Given the poor prognosis, he was transferred to palliative care and discharged for supportive management.

## Discussion

LC is a rare infiltrative subtype of PCNSL, characterized by diffuse white matter involvement without formation of a discrete mass lesion [3]. Its atypical imaging appearance frequently leads to misdiagnosis, most commonly as inflammatory demyelinating disease, resulting in delayed tissue diagnosis and initiation of appropriate therapy [9].

The characteristic imaging findings in LC, diffuse, bilateral T2-weighted and FLAIR hyperintensities with minimal or absent contrast enhancement and little to no diffusion restriction, reflect widespread perivascular and interstitial infiltration rather than focal tumor growth [5,6]. In contrast to classical PCNSL, disruption of the blood-brain barrier in LC is often limited, accounting for the lack of significant enhancement [3,9,12]. Diffusion restriction, when present, is typically mild or patchy, corresponding to the lower cellular density compared to focal tumor masses. These imaging features substantially overlap with those of inflammatory demyelinating disorders and diffuse infiltrating gliomas, particularly when optic pathway involvement is present [12].

Li et al. described key imaging features of LC, including involvement of more than three anatomical regions, diffuse white matter-predominant infiltration with extension to deep gray nuclei, minimal or absent enhancement, and lack of significant mass effect, in conjunction with histopathological evidence of angiocentric CD20-positive diffuse large B-cell lymphoma [3]. The imaging pattern in the present case closely mirrors these described features. The extensive, multiregional involvement involving both white matter and deep gray structures, along with optic pathway extension, was highly suggestive of LC. The combination of diffuse infiltration without mass effect and poor response to immunomodulatory therapy further favored a neoplastic process over demyelinating disease.

Corticosteroid responsiveness further complicates diagnosis. Steroids may induce transient clinical and radiologic improvement in lymphoma, thereby reinforcing an initial inflammatory diagnosis while simultaneously reducing the diagnostic yield of biopsy. Prior studies have demonstrated that corticosteroid pretreatment can significantly decrease biopsy sensitivity in both PCNSL and LC [2,11]. In the present case, prolonged exposure to corticosteroids and additional immunomodulatory therapies likely contributed to a delayed definitive diagnosis.

From a radiologic standpoint, certain features should prompt reconsideration of a demyelinating diagnosis. These include lack of sustained treatment response, progressive multifocal involvement of deep and subcortical white matter, extension into deep gray nuclei and brainstem, and absence of supportive CSF or serologic markers for inflammatory demyelination [8,11,12]. Early inclusion of LC in the differential diagnosis of diffuse white matter disease is therefore essential, particularly in steroid-refractory or clinically progressive cases [3,12].

The absence of CSF markers of demyelination, including oligoclonal bands, AQP4, and MOG antibodies, along with normal visual evoked potentials and progressive radiologic worsening despite immunotherapy, further supported a non-inflammatory etiology in this case. Other causes of diffuse white matter disease, including toxic, metabolic, infectious, and genetic leukoencephalopathies, were considered but deemed unlikely given the clinical context and negative workup.

Important radiologic differentials include optic nerve lymphoma and inflammatory optic neuropathies such as neuromyelitis optica spectrum disorder (NMOSD) and MOG-associated disease (MOGAD). Optic nerve lymphoma represents focal lymphomatous infiltration of the optic nerve, either in isolation or as part of systemic or primary central nervous system lymphoma [13]. Optic nerve lymphoma typically presents with fusiform nerve thickening, marked homogeneous enhancement, and diffusion restriction, and is usually localized rather than associated with diffuse parenchymal infiltration [13].

In contrast, NMOSD commonly demonstrates simultaneous or sequential involvement of bilateral optic nerves extending over at least half the length of the nerve with frequent chiasmatic involvement, along with characteristic brain lesions in AQP4-rich regions such as the periependymal surfaces, hypothalamus, and periaqueductal region [14,15]. The presence of AQP4 antibodies, along with a clinical history of relapsing optic neuritis and longitudinally extensive transverse myelitis, further supports the diagnosis.

MOGAD characteristically presents with bilateral, longitudinally extensive optic neuritis, often accompanied by optic disc swelling. Imaging commonly demonstrates involvement of the retrobulbar segment of the optic nerve, optic nerve sheath enhancement, or optic perineuritis [16]. Additional MRI features may include cortical or juxtacortical lesions and leptomeningeal enhancement [17]. MOGAD frequently affects children and young adults [18].

Definitive diagnosis of LC requires histopathological confirmation. Brain biopsy demonstrating angiocentric infiltration by CD20-positive malignant lymphoid cells remains the gold standard [5,12]. From a radiologic perspective, early recognition of suggestive imaging patterns and timely consideration of biopsy are crucial to avoid prolonged empiric immunosuppression and to facilitate appropriate oncologic management.

## Conclusions

This case illustrates a rare but important radiologic presentation of LC, emphasizing its potential to mimic inflammatory demyelinating disease. Progressive neurological symptoms, diffuse non-enhancing white matter abnormalities, optic pathway involvement, and lack of sustained response to immunomodulatory therapy should raise suspicion for an infiltrative neoplastic process. Awareness of this imaging phenotype and early consideration of biopsy are essential to minimize diagnostic delay and guide appropriate management.
